# Lusutrombopag as a substitute for platelet transfusion for thrombocytopenia associated with chronic liver disease in a patient undergoing endoscopic spinal surgery: A case report

**DOI:** 10.1097/MD.0000000000024094

**Published:** 2021-01-15

**Authors:** Takeshi Kaneko, Yuichi Takano, Katsuhiko Ishibashi

**Affiliations:** a Inanami Spine and Joint Hospital; b Iwai Orthopaedic Hospital, Tokyo, Japan.

**Keywords:** endoscopic surgery, lusutrombopag, micro-endoscopic laminectomy, minimally invasive spine surgery

## Abstract

**Introduction:**

Bleeding may interfere with the visual field and create difficulties in performing the intended treatment, especially in operations involving a small working space such as endoscopic spinal surgery. Therefore, it is important to reduce the risk of bleeding before surgery.

**Patient concerns:**

A 76-year-old female presented with a history of right anterior thigh pain along the L3 dermatome for 3-years, following a L3 compression fracture. In addition, the patient had developed autoimmune hepatitis at 50 years of age, and the platelet count on laboratory blood collection was 78 × 10^9^/L.

**Diagnosis:**

Magnetic resonance (MR) images showed a narrowed foramen at the L3–4 level. L3 nerve block was effective. L3 foraminal-stenosis was suspected.

**Interventions:**

Micro-endoscopic laminectomy (MEL) for foraminal decompression was planned due to possible L3 nerve root compression. Lusutrombopag, a thrombopoietin (TPO) receptor agonist, was orally administered for 7 days starting 7 days preoperatively to address the risks of bleeding.

**Outcomes:**

The patient successfully underwent MEL without any adverse events or complications.

**Conclusion:**

The results obtained from the use of lusutrombopag suggested that safety measures could be implemented preoperatively, and that lusutrombopag may be a useful supplemental drug for minimally invasive treatment of patients with cirrhosis and thrombocytopenia.

## 1 Introduction

Thrombocytopenia is a common abnormality in patients with chronic liver disease (CLD). Approximately 10% of patients with cirrhosis are known to show a severe decrease in platelet count that falls below 50 × 10^9^/L.^[1]^ Thrombocytopenia can cause bleeding and may become an obstacle in performing the desired treatment. Because the surgical field is narrow in minimally invasive surgery, bleeding may disturb its visualization, especially for endoscopic spinal surgery. Therefore, potential risks during surgery can be greatly reduced if the risk of bleeding can be prevented prior to surgery.

We report a case of thrombocytopenic cirrhosis wherein a preoperative platelet increase was confirmed with the oral administration of lusutrombopag, a thrombopoietin (TPO) receptor agonist. The patient successfully underwent spine surgery without any adverse events or complications. Written informed consent was obtained from a patient for the publication of this case report. Our institution does not require ethical approval for reporting individual cases.

## 2 Case report

**Figure 1:**
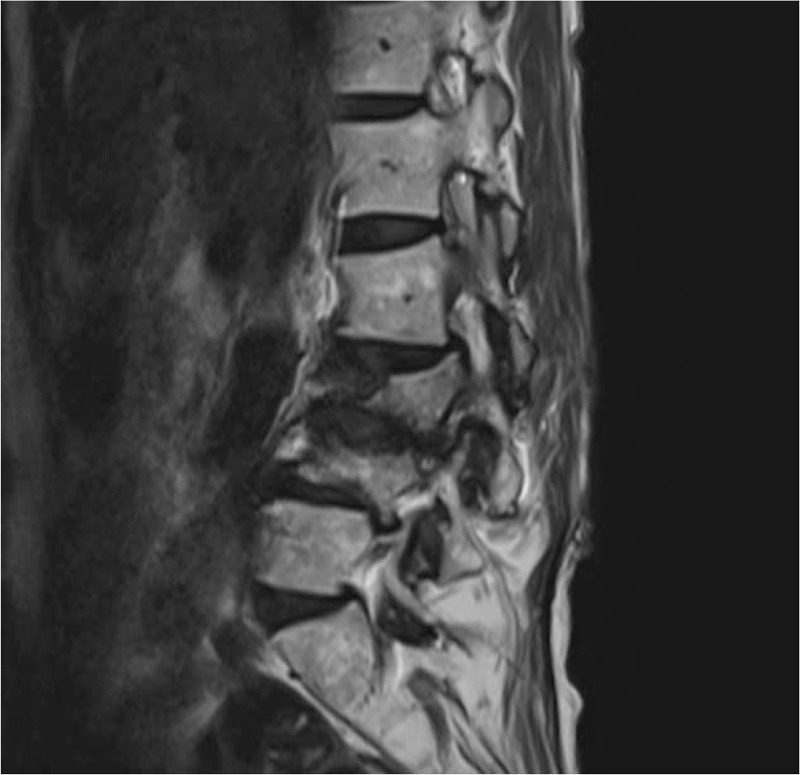
Sagittal image on MRI shows L3 compression fracture and L3 nerve root compression. The patient underwent a MEL for foraminal encroachment.

**Figure 2:**
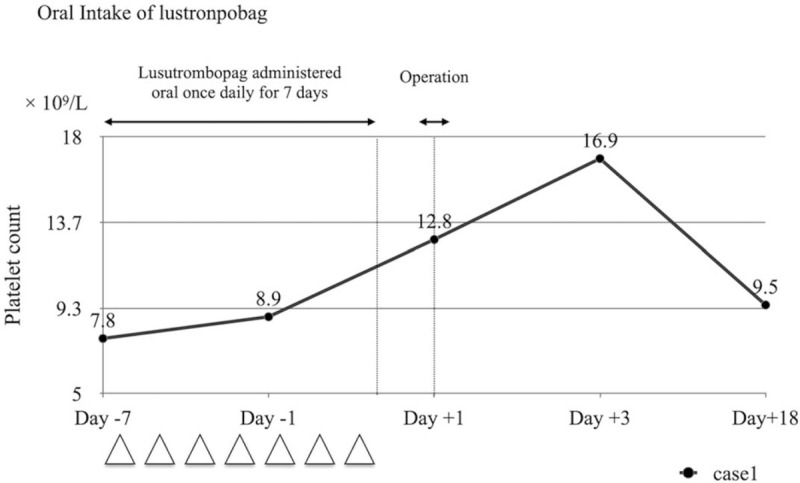
Changes in platelet count after administration of lusutrombopag. The platelet count exhibited a gradual increase, including the postoperative period. At 1 day preoperatively, the platelet count increased to 89 × 10^9^/L, enabling surgical intervention without difficulties and helped in maintaining perioperative hemostasis.

A 76-year-old female presented with a history of right anterior thigh pain along the L3 dermatome for 3-years following an L3 compression fracture. Magnetic resonance (MR) images showed a narrowed foramen at the L3–4 level (Fig. 1). L3 nerve block was effective. Micro-endoscopic laminectomy (MEL) for foraminal decompression was planned due to possible L3 nerve root compression. However, the patient had developed autoimmune hepatitis at 50 years of age, and the platelet count on laboratory blood collection was 78 × 10^9^/L. Due to concerns of bleeding, lusutrombopag was oral administered 3 mg/day for 7 days from 7 days preoperatively. The platelet count showed an increase at 8.9 × 10^9^/L 2 days before surgery. The platelet count immediately before the operation was 128 × 10^9^/L, and MEL was performed on day 8 after initiation of medication (Fig. 2). There were no intraoperative events that hindered the operation due to bleeding. The drainage volume was 50 cm^3^. No postoperative lower limb pain or increased severity of paralysis was observed. A sufficient amount of platelets was confirmed with a platelet count of 16.9 × 10^9^/L 2 days after surgery. Therefore, platelet transfusions were not performed during the perioperative period. The patient was discharged from the hospital at 7 days postoperatively without any adverse events or side effects due to intraoperative bleeding. Eighteen days after the final administration, the platelet count decreased to 9.5 × 10^9^/L.

## 3 Discussion

Endoscopic spinal surgery involves a particularly small surgical field, even among minimally invasive techniques. Therefore, bleeding may increase perioperative risks due to difficulties in securing a visual field.

In chronic liver diseases, platelet count may decrease due to acceleration of platelet destruction and decreased TPO production as a result of hypersplenic thrombocytopenia, particularly for patients with cirrhosis.^[2]^ TPO is the main hematopoietic factor produced in the liver and promotes platelet production. Lusutrombopag is an orally administered, small molecular weight TPO receptor agonist that was first approved in Japan. This agent is used to improve CLD-related thrombocytopenia in patients planning to undergo an invasive procedure.^[3]^ It is believed to act on the transmembrane region of the TPO receptor expressed in megakaryocytes, which proliferate and differentiate megakaryocyte progenitor cells through a signaling pathway to produce platelets.^[4]^

Platelets play an important role in hemostatic processes.^[1]^ These processes include primary hemostasis, in which platelets aggregate at the site of injury to induce thrombus formation, and secondary hemostasis, in which coagulation factors are activated and a fibrin mesh is formed from fibrinogen. In both of these processes, platelets play a crucial role.^[5]^ Therefore, it is considered important to confirm the platelet count prior to surgery. When performing surgery, it is said that there is a high risk of bleeding if the platelet count is 50 × 10^9^/L or less. In our present case, the platelet count was 7.8 × 10^9^/L, which was less than the required threshold of 50 × 10^9^/L, and hence the patient required an increase in platelet count. However, Friedman^[6]^ recommended to maintain the platelet count above 100 × 10^9^/L during the perioperative period for total hip arthroplasty (THA).

Although the risk of administering this drug includes thrombosis that has been reported in its Phase II and Phase III trials,^[7]^ no postoperative thrombosis was observed. However, prone positioning is a common position in spinal surgery and may create abdominal compression. For this reason, the positioning of the body is usually supported by the chest and iliac bones with a four-point frame to allow decompression. In cases of severe obesity, however, abdominal compression due to fat should be avoided.

In this report, an increased preoperative platelet count was confirmed in a patient with thrombocytopenic cirrhosis after lusutrombopag administration. The patient successfully underwent surgery without difficulties in perioperative hemostasis management. The results obtained from the use of lusutrombopag suggested that safety measures could be implemented preoperatively, and that lusutrombopag may be a useful supplemental drug for the minimally invasive treatment of patients with cirrhosis and thrombocytopenia.

## Author contributions

**Data curation:** Katsuhiko Ishibashi.

**Supervision:** Yuichi Takano.

## Abbreviations

Abbreviations: CLD = chronic liver disease, MEL = micro-endoscopic laminectomy, MR = magnetic resonance, THA = total hip arthroplasty, TPO = thrombopoietin.
